# *Dinophysis acuta* in Scottish Coastal Waters and Its Influence on Diarrhetic Shellfish Toxin Profiles

**DOI:** 10.3390/toxins10100399

**Published:** 2018-09-28

**Authors:** Sarah C. Swan, Andrew D. Turner, Eileen Bresnan, Callum Whyte, Ruth F. Paterson, Sharon McNeill, Elaine Mitchell, Keith Davidson

**Affiliations:** 1 Scottish Association for Marine Science, Scottish Marine Institute, Oban, Argyll PA37 1QA, UK; callum.whyte@sams.ac.uk (C.W.); ruthflo.paterson@gmail.com (R.F.P.); sharon.mcneill@sams.ac.uk (S.M.); elaine.mitchell@sams.ac.uk (E.M.); keith.davidson@sams.ac.uk (K.D.); 2 Centre for Environment, Fisheries & Aquaculture Science, The Nothe, Barrack Road, Weymouth, Dorset DT4 8UB, UK; andrew.turner@cefas.co.uk; 3 Marine Scotland Science, Marine Laboratory, 375 Victoria Road, Aberdeen AB11 9DB, UK; eileen.bresnan@gov.scot

**Keywords:** *Dinophysis*, HAB monitoring, DSP toxins, aquaculture, shellfish toxicity, human health, time-series, seasonality, Scotland

## Abstract

Diarrhetic shellfish toxins produced by the dinoflagellate genus *Dinophysis* are a major problem for the shellfish industry worldwide. Separate species of the genus have been associated with the production of different analogues of the okadaic acid group of toxins. To evaluate the spatial and temporal variability of *Dinophysis* species and toxins in the important shellfish-harvesting region of the Scottish west coast, we analysed data collected from 1996 to 2017 in two contrasting locations: Loch Ewe and the Clyde Sea. Seasonal studies were also undertaken, in Loch Ewe in both 2001 and 2002, and in the Clyde in 2015. *Dinophysis acuminata* was present throughout the growing season during every year of the study, with blooms typically occurring between May and September at both locations. The appearance of *D. acuta* was interannually sporadic and, when present, was most abundant in the late summer and autumn. The Clyde field study in 2015 indicated the importance of a temperature front in the formation of a *D. acuta* bloom. A shift in toxin profiles of common mussels (*Mytilus edulis*) tested during regulatory monitoring was evident, with a proportional decrease in okadaic acid (OA) and dinophysistoxin-1 (DTX1) and an increase in dinophysistoxin-2 (DTX2) occurring when *D. acuta* became dominant. Routine enumeration of *Dinophysis* to species level could provide early warning of potential contamination of shellfish with DTX2 and thus determine the choice of the most suitable kit for effective end-product testing.

## 1. Introduction

Naturally occurring harmful algal blooms (HABs) are known to have an adverse effect on shellfish industries worldwide, with toxic contamination of shellfish from these events potentially resulting in both human illness and a detrimental impact on the often-fragile economies of rural areas [1]. Despite regulatory monitoring, the accumulation of toxins in shellfish has led to occasional reports of sickness, with relatively infrequent outbreaks of phytoplankton-generated Diarrhetic Shellfish Poisoning (DSP) around Europe and elsewhere since it was first reported from The Netherlands in the 1960s [2,3,4]. In the UK, DSP was associated with the ingestion of imported mussels (*Mytilus* spp.) in 1994 and with UK common (blue) mussels (*Mytilus edulis*) obtained from an unauthorised site in 1997 [5,6,7]. Common (blue) mussels harvested in Scotland were also linked to 159 cases of DSP in 2006 [8,9] and a further 70 reported cases in 2013 [10]. While these events are relatively few in number, their impact on consumer confidence and industry sustainability is significant.

To mitigate the risk of human illness caused by the consumption of contaminated shellfish, European Union regulations require EU Member States to have regulatory programmes in place to monitor the presence of both marine biotoxins in shellfish production areas and the causative phytoplankton [11]. In the UK, toxin testing of shellfish tissue is supported by the analysis of seawater samples for the presence of toxin-producing phytoplankton and results delivered by the monitoring programmes are used to make decisions regarding the opening and closure of classified shellfish harvesting areas. In Scotland, this information is also used by the aquaculture industry to make informed decisions, following guidance issued to harvesters by Food Standards Scotland (FSS) in 2014 [12], which may lead to either an increase in end-product testing to ensure the safety of shellfish placed on the market, or a voluntary cessation of harvesting.

Between 2012 and 2017, an average of over 2700 tests have been carried out each year on bivalves, around 70% of which were common (blue) mussels, collected from approximately 80 classified shellfish harvesting areas in Scottish coastal waters. One of the main causes for concern is the presence of lipophilic toxins, some of which cause DSP. The accumulation of these toxins in shellfish is a major problem for the aquaculture industry in Scotland. Since routine monitoring for these toxins began in 1998 using the mouse bioassay (MBA) [13], extensive harvesting closures that can last for several months in some areas have been enforced. The DSP toxins include okadaic acid (OA) and the dinophysis toxins, dinophysistoxin-1 (DTX1) and dinophysistoxin-2 (DTX2), henceforth referred to as the OA group toxins. The group collectively known as DTX3 are derivatives of OA, DTX1 and DTX2, esterified with saturated and unsaturated fatty acids [9] and the chemical structure of these toxins is described in detail elsewhere [9,14]. The relative potency of the toxins within the OA group differs, with the toxic potential of DTX1 being similar to that of OA, although both are more toxic than DTX2 [9]. Hence, a Toxicity Equivalency Factor of 0.6 is used for DTX2. The use of the MBA method meant that individual OA group toxins could not be routinely identified and previous information on the presence of these toxins came from research studies using LC-MS [13,15]. The introduction of LC-MS/MS methodology into the regulatory toxin testing in July 2011 provided the scope to assess the presence of this toxin group on a regional and temporal scale [16]. The OA group toxins are responsible for most of the toxin contamination in Scottish bivalves and the maximum permitted level (MPL) in harvestable shellfish is 160 µg okadaic acid equivalent per kg shellfish flesh (OA eq./kg) [9]. The percentage of shellfish tissue samples with OA group toxicity reported above the regulatory limit fluctuates by year [16] and between 2012 and 2017, it varied between 2.2% (in 2017) and 11.5% (in 2013), with annual maximum amounts of total OA group equivalent toxicity ranging from 694 µg OA eq./kg in 2017 to 4993 µg OA eq./kg in 2013. The exceptionally high toxin maximum from a production area in the Shetland Islands in 2013 resulted in an outbreak of DSP [10].

A number of causative phytoplankton species are associated with lipophilic toxins and those connected with the OA group toxins belong to the order Dinophysiales and include the genera *Dinophysis* Ehrenberg and *Phalacroma* Stein. Algal cells in this order are routinely identified using light microscopy in regulatory monitoring programmes. In Scottish waters they are currently reported as total *Dinophysis* spp. with an ‘alert’ threshold set at 100 cells/L, to ensure testing of shellfish for the presence of biotoxins. The species concept within *Dinophysis* is not clearly defined [17,18] and a certain amount of gradation in character traits can lead to morphological ambiguity. Differences in cell shape and size have been attributed to geographic variation, environmental selection, feeding behaviour and life cycle [19]. Stern et al. [18] examined the genetic sequences of cells from Scottish coastal waters with morphologies that appeared to belong to the *D. acuminata* complex and identified the presence of both *D. acuminata* Claparéde & Lachmann and *D. ovum* and also confirmed the dominance of *D. acuminata* during late spring/summer. The other main species observed is *D. acuta* Ehrenberg but with considerable interannual variability in abundance [1]. Blooms dominated by *D. acuta* have occasionally been recorded, as was the case for 2001 and 2002 in Scapa Bay in Orkney [13]. *Dinophysis dens* is sometimes observed at low concentrations in blooms of *D. acuta* and is now regarded as a life-stage of *D. acuta* [20,21]. *Phalacroma rotundatum* is also regularly detected around the Scottish coast but again at low concentrations rarely exceeding 100 cells/L. Although it has been found to contain toxins, there is some evidence that it may not be a toxin-producer itself but may instead act as a vector [22].

Another known producer of diarrhetic shellfish toxins (DSTs) is the benthic dinoflagellate *Prorocentrum lima*. This species is detected more often in the sandy sediments of shallow bays where oyster cultivation takes place, although it can also grow epiphytically [23]. *Prorocentrum lima* is recorded sporadically in integrated water column samples but cell counts are likely to be underestimated using this method and it is generally more frequently observed in samples obtained by bucket. Analysis of the data obtained through the Scottish monitoring programme failed to establish a clear link between the presence of DSP toxins in bivalve molluscs and the abundance of *P. lima* (Scottish Association for Marine Science (SAMS) unpublished data). Hence this study is focused on the apparent link between DSTs in shellfish and the presence of *Dinophysis*.

**Figure 1:**
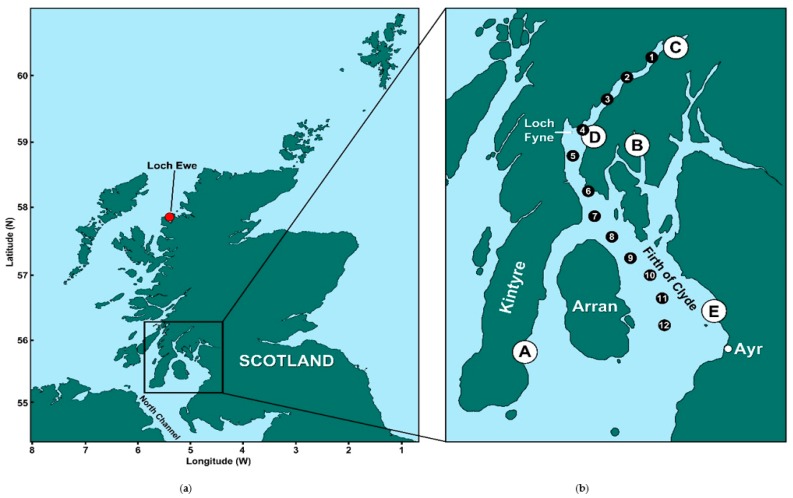
Maps of Scotland (**a**) and the study area showing the location of the Firth of Clyde and the official control monitoring sites for phytoplankton and shellfish (**b**), indicated by the white circles (A = Campbeltown Loch, B = Loch Striven, C = Loch Fyne: Ardkinglas, D = Loch Fyne: Otter Ferry and E = Barassie). Mussels are harvested at sites A, B and C, Pacific oysters at site D and razor clams at site E. The black circles (numbered 1 to 12) show the location of the additional phytoplankton samples obtained from the research survey conducted in early September 2015. The location of the long-term monitoring site at Loch Ewe is indicated in (**a**).

In order to understand variability within the *Dinophysis* population and its influence on toxin accumulation in bivalve molluscs, this study was undertaken to investigate the annual and seasonal variation of *Dinophysis* spp. and associated toxins in two important shellfish harvesting regions on the west coast of Scotland, Loch Ewe and the Firth of Clyde (Figure 1). Hence, the risks to human health associated with changes in species composition of blooms can be evaluated.

## 2. Results

### 2.1. Loch Ewe

#### 2.1.1. *Dinophysis* Abundance

**Figure 2:**
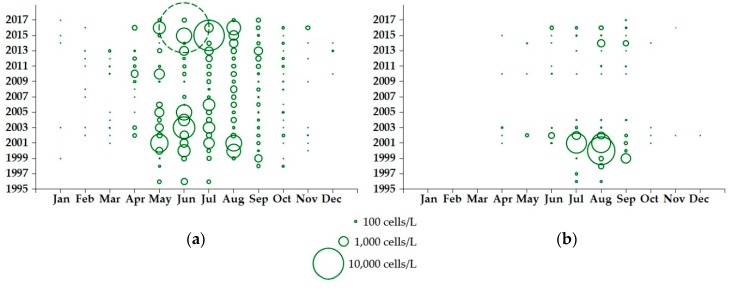
Maximum abundance by month for (**a**) *D. acuminata* and (**b**) *D. acuta* in phytoplankton samples obtained from Loch Ewe (NW Scotland) between 1996 and 2017, based on the analysis of 1797 records. The dashed circle in (**a**) represents a dense bloom of *D. acuminata* recorded in Loch Ewe in 2016 (24,340 cells/L).

The abundance of *Dinophysis* in Loch Ewe between 1996 and 2017 varied considerably by month and between years. *Dinophysis acuminata* was recorded during the phytoplankton growing season (spring/summer) every year and in some instances during the winter months as well. Counts at or exceeding the ‘alert’ threshold that triggers shellfish toxin testing (100 cells/L) were obtained from March to November but the species was typically most abundant between May and August (Figure 2a). By contrast, *D. acuta* was never recorded between January and March, was detected above the ‘alert’ threshold from May to September but was most abundant between July and September (Figure 2b), with *D. acuminata* cells often present in the community at the same time. Bloom densities were not consistent between years, with *D. acuminata* typically recorded at maximum annual densities of less than 3000 cells/L but with some notable blooms in 2003, 2015 and 2016, when cell densities reached 4940 cells/L, 9540 cells/L and 24,340 cells/L, respectively. *Dinophysis acuta* was generally much less abundant, apart from an increase in cell densities between 1999 and 2002, including an exceptional bloom of 8040 cells/L recorded in August 2000.

#### 2.1.2. Toxin Concentration in Shellfish

**Figure 3:**
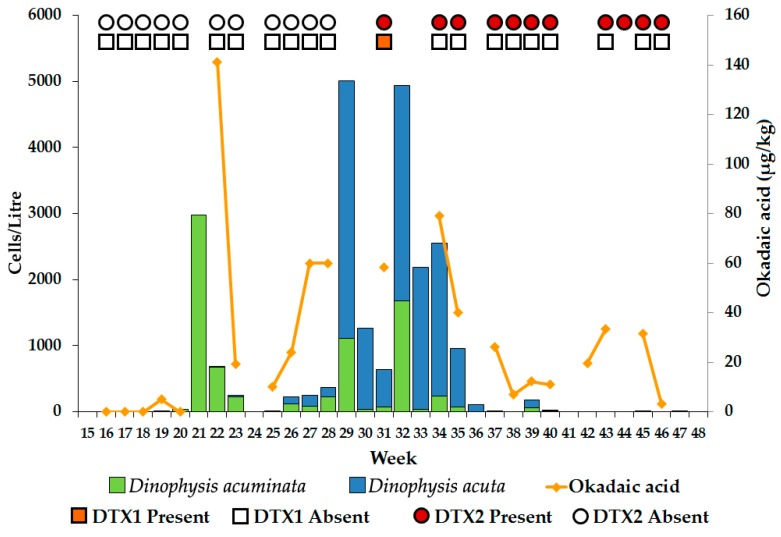
Stacked bar chart showing the abundance of *D. acuminata* and *D. acuta* and okadaic acid in common mussels from Loch Ewe during 2001. Cell counts were averaged for triplicate sub-samples every week. DTX1 and DTX2 were recorded as being either present or absent and are indicated by symbols.

**Figure 4:**
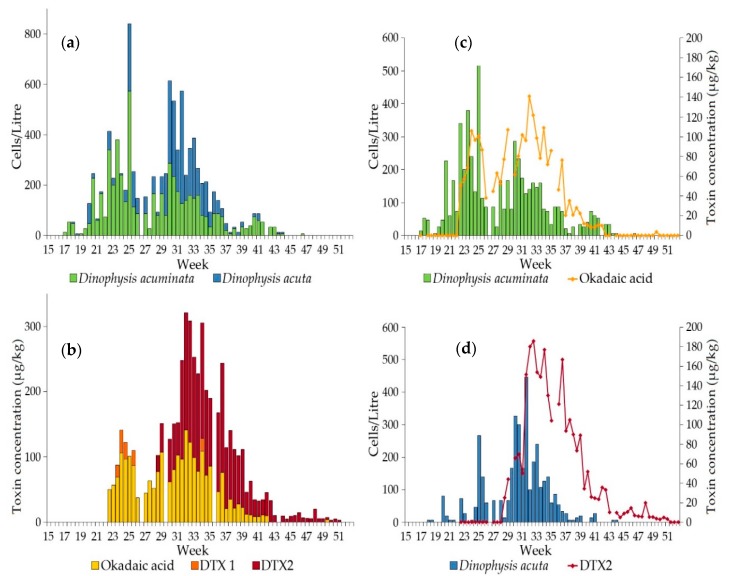
Abundances of *D. acuminata* and *D. acuta* in Loch Ewe during 2002 are shown in (**a**). Cell counts were averaged from triplicate sub-samples collected twice a week. Toxin concentrations in common mussels for the corresponding weeks are shown in (**b**). The relationships between *D. acuminata* and OA and between *D. acuta* and DTX2 are shown in (**c**,**d**), respectively.

LC-MS analysis was carried out on samples of common mussel tissue obtained from Loch Ewe in 2001 and 2002, coinciding with blooms of *Dinophysis*. For the 2001 investigation, DTX1 and DTX2 were recorded as being either present or absent, although OA was quantified. Weekly sampling occurred in 2001 and OA group toxins were detected in mussels, with the concentration of OA reaching a maximum value of 141 µg/kg in early June (week 22), associated with a bloom of *D. acuminata* of density 2980 cells/L in the preceding week (Figure 3). DTX2 was recorded as absent until early August (week 31), following a bloom of *D. acuta* at a density of 3900 cells/L in mid July (week 29). DTX1 was only detected on one occasion, also in week 31. In 2002 the sampling frequency in Loch Ewe was increased to twice a week. Both species of *Dinophysis* were less abundant, although *D. acuminata* levels began to increase from around mid May, reaching a maximum value of 573 cells/L in mid June (week 25), whereas *D. acuta* reached a maximum abundance of 447 cells/L in early August (latter half of week 31) (Figure 4a). Okadaic acid was detected in mussels in every week continuously from late May until mid October but DTX1 was infrequently detected and mostly occurred in June when *D. acuminata* was the dominant species (Figure 4b). DTX2 was more associated with *D. acuta* (Figure 4d) and was absent until mid July (latter half of week 28), reaching a maximum value of 186 µg/kg one week after the *D. acuta* bloom peak of 447 cells/L. The apparent delay in toxin accumulation in the mussels following the *Dinophysis* blooms (Figure 4c,d) was investigated using a non-parametric Spearman’s Rank-Order Correlation. The relationships between the individual toxins, OA and DTX2 and the abundance of both *D. acuminata* and *D. acuta* were explored over time, with lags of between 0 and 3 weeks at half-weekly intervals. A strong positive correlation was identified between *D. acuminata* and OA throughout the bloom period, reaching a maximum value with a two-week lag (*r*_s_ = 0.815, *p* < 0.001). We found no significant correlation between *D. acuminata* and DTX2. A strong positive correlation was also identified between *D. acuta* and OA throughout the bloom period but as both *D. acuminata* and *D. acuta* were present at the same time throughout most of the summer, it is difficult to discriminate the toxin contribution from each individual species. However, *D. acuta* was significantly correlated with DTX2, reaching a maximum value with a lag of 1.5 weeks (*r*_s_ = 0.569, *p* < 0.001).

### 2.2. Firth of Clyde

#### 2.2.1. *Dinophysis* Abundance

**Figure 5:**
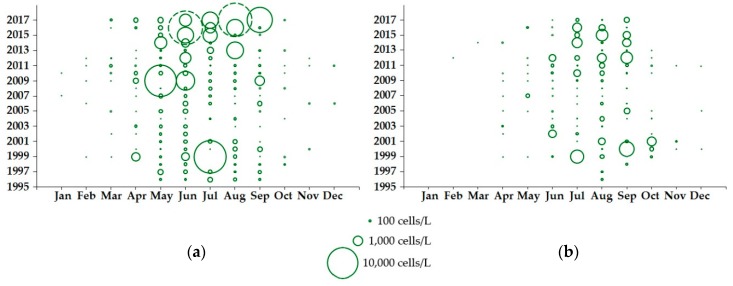
Maximum abundance of (**a**) *D. acuminata* and (**b**) *D. acuta* recorded at monitoring sites around the Firth of Clyde between 1996 and 2017, based on the analysis of 1001 records from eight sampling locations. The dashed circles in (**a**) represent dense blooms of *D. acuminata* observed in Loch Fyne: Ardkinglas (Site C in Figure 1b) in 2016 (85,760 cells/L) and 2017 (180,289 cells/L).

Time-series data collected between 1996 and 2017 from eight sampling locations around the Firth of Clyde and associated sea lochs showed a greater variability in *D. acuminata* abundance (Figure 5a). As in Loch Ewe, this species was detected in every month of the year, although this was not the case for every year and with above ‘alert’ threshold events from March to October. Some exceptionally dense blooms were observed in 1999, 2009, 2016 and 2017, with recorded cell densities of 13,860 cells/L, 13,260 cells/L, 85,760 cells/L and 180,289 cells/L, respectively. Apart from the 1999 bloom recorded in Loch Striven (Figure 1b, site B), the other dense blooms were all detected in upper Loch Fyne: Ardkinglas (Figure 1b, site C). *Dinophysis acuta* in the Firth of Clyde (Figure 5b) showed a similar profile to that in Loch Ewe, with cell counts also highest between July and September. Interannual variability was evident, with the densest *D. acuta* blooms occurring between 1999 and 2002 and again between 2012 and 2016, coinciding with an increase in *D. acuta* blooms further north in Loch Ewe.

**Figure 6:**
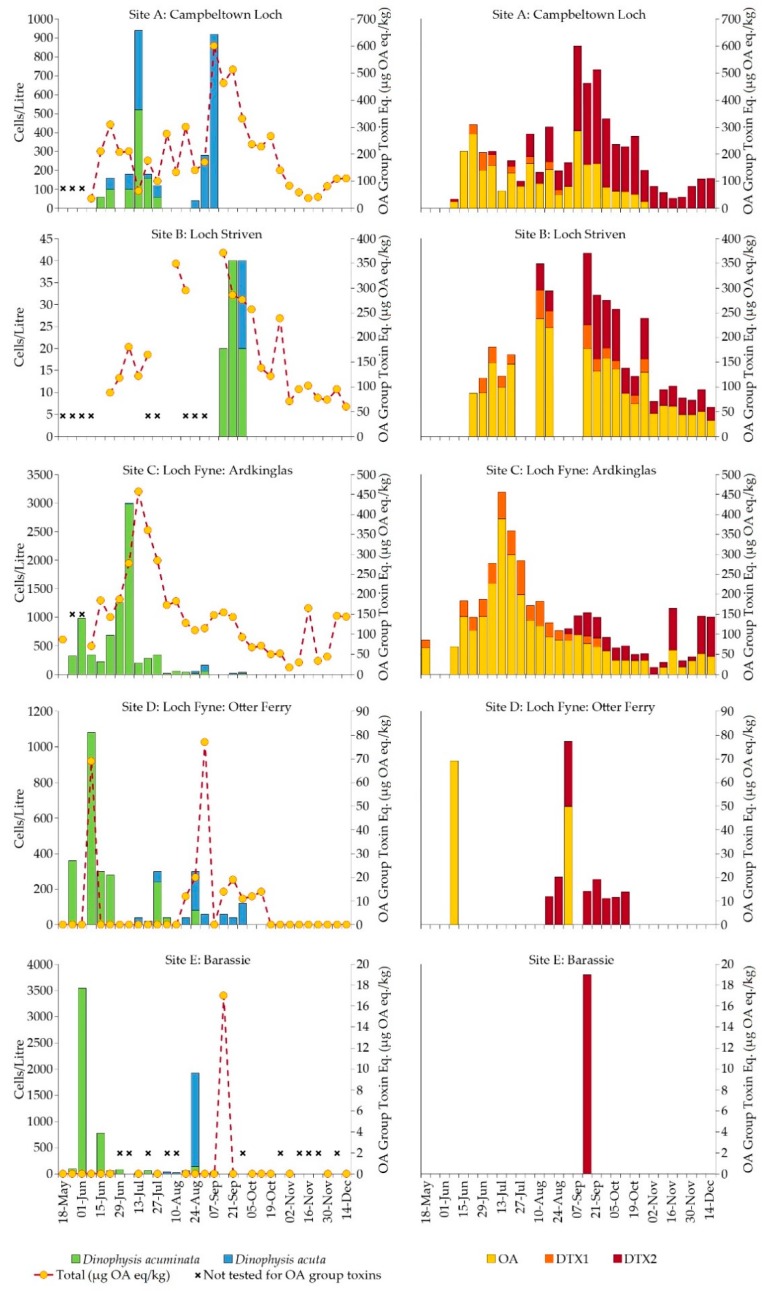
Left hand column shows the abundance of *D. acuminata* and *D. acuta* at official control monitoring sites and total OA group toxin equivalent (yellow circles) detected in shellfish between May and December 2015. Right hand column shows the contribution of OA, DTX1 and DTX2 (including esters) to the total OA group value (Toxicity Equivalency Factor of 0.6 applied to DTX2). No testing for lipophilic toxins was performed in late May and early June at the three mussel sites (A, B and C) due to the presence of paralytic shellfish toxins above the regulatory limit.

A detailed examination of data obtained from the regulatory monitoring sites around the Firth of Clyde in 2015 also showed distinct patterns of abundance and seasonality for *D. acuminata* and *D. acuta* (Figure 6). Phytoplankton counts identified a bloom of predominantly *D. acuminata* widespread throughout the area from late May into mid July 2015. The highest recorded densities were 3540 cells/L at Barassie (Site E) on 2 June, 1080 cells/L at Loch Fyne: Otter Ferry (Site D) on 9 June and 2980 cells/L further up Loch Fyne at Ardkinglas (Site C) on 7 July. During this early part of summer at most of the sites, the *Dinophysis* population was exclusively *D. acuminata*. However, a mixed bloom of *D. acuminata* and *D. acuta* was observed in Campbeltown Loch (Site A) on 13 July, with cell abundances of 520 and 420 cells/L, respectively. Subsequently, *D. acuta* dominated the *Dinophysis* in Campbeltown Loch, reaching a maximum of 920 cells/L on 7 September. An increase in the abundance of *D. acuta* was also noted at all the other monitoring sites from around mid August, with a maximum bloom density of 1780 cells/L detected at Barassie on 25 August. Although regulatory phytoplankton monitoring did not begin in Loch Striven (Site B) until 15 September and *Dinophysis* counts throughout the whole area were relatively low by this time, many empty *D. acuta* theca were observed, indicating that the bloom had extended into Loch Striven in the preceding weeks.

**Figure 7:**
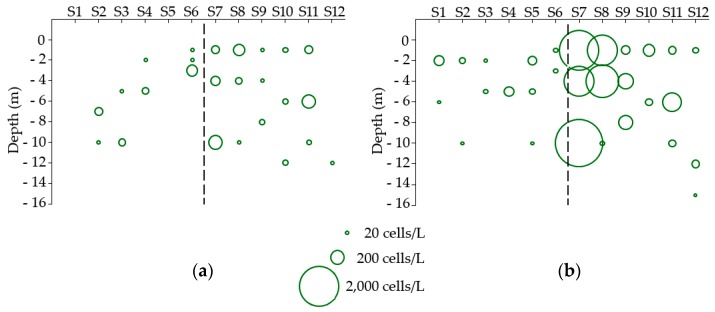
Abundance of (**a**) *D. acuminata* and (**b**) *D. acuta* in phytoplankton samples obtained from the research survey in early September. Stations S1 to S6 are in Loch Fyne and stations S7 to S12 are the more open waters of the Firth of Clyde (see Figure 1b). The temperature front between stations S6 and S7 is represented by a dashed line.

Data from the September 2015 research cruise in the Firth of Clyde were consistent with the phytoplankton data from the regulatory monitoring sites. Figure 7a shows that *D. acuminata* was not particularly abundant in the samples from the cruise transect, with a maximum density of 200 cells/L recorded at Stations S7 and S11, to the seaward side of a marked temperature front between stations S6 and S7 [24]. The front was characterized by a body of cooler water located between Stations S4 and S6, with temperatures either side of the front being, on average, approximately 1 °C higher. By contrast, densities of *D. acuta* were more than ten times greater, with a peak abundance of 2840 cells/L at Station S7 near the mouth of Loch Fyne. There were no depth-related trends for either species but the highest densities of *D. acuta* were found in Stations S7 and S8 (Figure 7b), also coincident with the temperature front and on the seaward side. Abundance of *D. acuta* did not exceed 140 cells/L within Loch Fyne itself (Stations S1–S6).

#### 2.2.2. Okadaic Acid Group Equivalent Toxicity and Toxin Concentration in Shellfish

Figure 6 shows the variability in overall OA group toxicity in shellfish tissue collected from the regulatory monitoring sites around the Firth of Clyde between May and December 2015. The highest total toxin equivalent levels were recorded in mussels, with a value of 601 µg OA eq./kg reported in samples collected from Campbeltown Loch (Site A) on 7 September, coinciding with the peak abundance of *D. acuta* recorded at this site. A value of 457 µg OA eq./kg was reported, also in mussels, from Loch Fyne: Ardkinglas (Site C) a week after the dense bloom of 2980 cells/L of *D. acuminata* on 7 July. Okadaic acid group equivalent toxicity was lower in Pacific oysters and razor clams and the period of contamination was shorter, compared with that of the mussels. Two distinct OA group peaks were observed in Pacific oysters from Loch Fyne: Otter Ferry (Site D), the first peak of 69 µg OA eq./kg coinciding with the *D. acuminata* bloom of 1080 cells/L on 9 June and the second peak of 77 µg OA eq./kg on 1 September, following an increase in *D. acuta* in the preceding week. The maximum reported OA group equivalent toxin value in razor clams from Barassie (Site E) was much lower than in other shellfish species. Okadaic acid group toxicity at 17 µg OA eq./kg was detected in a razor clam sample collected on 14 September, three weeks after a bloom of density 1980 cells/L that was composed of about 90% *D. acuta*. The earlier *D. acuminata* bloom at this site of 3540 cells/L recorded on 2 June did not appear to have any associated toxin contamination of shellfish. The detection of OA group toxins in mussels, including those from other biotoxin monitoring sites around the Firth of Clyde, continued for an extended period throughout autumn and into the winter months despite the absence of any causative organism by this time.

**Figure 8:**
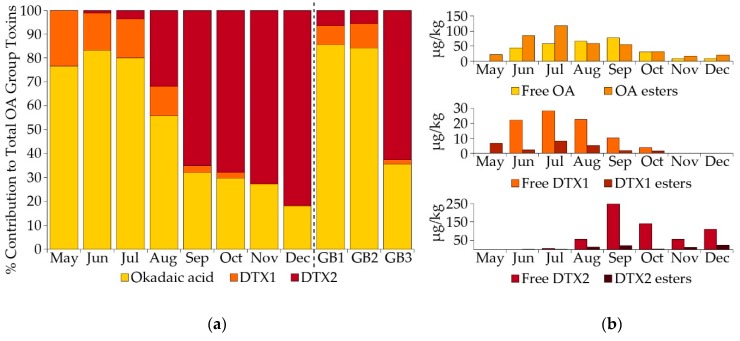
Mean percentage contribution of OA analogues and derivatives to total OA group toxins in mussels from sites A, B and C in the Firth of Clyde between May and December 2015 (**a**). For comparative purposes, the typical profiles obtained for mussels from Great Britain (GB1, GB2 and GB3) in the study by Johnson et al. [26] are also shown. (**b**) shows the proportion of free and esterified forms of each OA group toxin detected in the Firth of Clyde mussels.

The proportion of each toxin analogue within the OA group changed over time for the three mussel sites A, B and C (Figure 8a). June mussel samples contained approximately 83% OA in both free and ester form, which decreased over time but was still present in December samples, contributing approximately 18% to the total OA group toxins by this time. Low levels of DTX1 were also recorded, with a maximum contribution of about 23% during May, declining to below the reporting limit by November. Concentrations of total OA and total DTX1 were detected at maximum values of 389 µg/kg and 85 µg/kg, respectively from mussels collected at Loch Fyne: Ardkinglas in mid and late July 2015 (Figure 6). These elevated levels of OA and DTX1 appeared to follow the bloom of *D. acuminata* of density 2980 cells/L in early July. The DTX2 contribution increased from approximately 1% of the total OA group toxins in June to almost 82% by December (Figure 8a). The highest recorded amount of DTX2 was 581 µg/kg (with Toxicity Equivalency Factor value of 349 µg/kg), detected in mussels from Campbeltown Loch in late September 2015 (Figure 6). A greater proportion of the OA detected between May and July was in ester form (Figure 8b), with a ratio of 2:1 for esters:free but more similar proportions of free OA and OA esters were present in samples between August and October. Both DTX1 and DTX2 were detected mostly in free forms, except in May when DTX1 was all in ester form. Although low levels of pectenotoxins (PTXs) have previously been detected in mussels from Loch Fyne in 2009 during a bloom of predominantly *D. acuminata* [25], none were found in this study.

## 3. Discussion

### 3.1. Environmental Influences on Seasonality and Abundance of Dinophysis

Both *Dinophysis acuminata* and *D. acuta* are regularly detected in the coastal waters of the North East Atlantic and the presence of these species, even at relatively low concentrations, results in a high incidence of shellfish contaminated with DSP toxins along the European Atlantic coast [2,27,28]. In Scottish coastal waters, maximum bloom densities of *Dinophysis* surveyed through the official control regulatory monitoring programme over the past 20 years have typically been in the region of 2000–10,000 cells/L, with infrequent observations of much denser patches, often in relatively enclosed areas. *Dinophysis* tends to be most abundant between May and September, prior to a late autumn decrease in line with the reduction in light levels associated with winter in temperate latitudes. The greatest *Dinophysis* cell densities in Scottish waters that usually occur between June and August are mainly composed of cells belonging to the *D. acuminata* complex. *Dinophysis acuta* blooms are observed less frequently than those of *D. acuminata* and tend to occur later in the year, typically from July onwards.

Our detailed analysis of the Clyde Sea sites and Loch Ewe conducted here concurs with the above qualitative assessment, indicating a dominance of *D. acuminata* in spring/summer with, in some years only, a switch to *D. acuta* dominance in late summer or autumn. Such temporal patterns have been observed elsewhere, for example along the Iberian shelf where the development of *D. acuminata* blooms usually begin in early March with the growth season extending until the autumnal transition from upwelling to downwelling [27,28,29]. In this region, *D. acuta* tends to occur in late summer-early autumn, although not every year, and has been associated with exceptionally hot summers [30] followed by a brief period of upwelling activity, leading to highly stratified conditions [2,21,28,31]. Blooms of *D. acuta* occurred between 1999 and 2002 in both Loch Ewe and around the Firth of Clyde with further occurrences in the Clyde and, to a lesser extent, Loch Ewe between 2014 and 2016. While the patterns of upwelling/downwelling that occur in Iberia are not a characteristic of Scottish waters, *D. acuta* blooms typically occur when seasonal stratification of the water column is likely [32,33].

Long-term variation in *Dinophysis* may be linked to climate [34,35]. Based on a modelling study, Gobler et al. [36] proposed that ocean warming had increased the growth rate and duration of the bloom season of *D. acuminata* over the period 1982–2006 in the North East Atlantic around the UK. However, following analysis of data from the Continuous Plankton Recorder (CPR) from the region, Dees et al. [37] did not find any increase in the number or annual duration of *Dinophysis* blooms. While the greatest number of *D. acuminata* above threshold counts occurred in each of the last three years (2015–2017), our results (Figure 2 and Figure 5), albeit over a shorter timescale than that modelled by Gobler et al. [36], concur with the conclusion that there is no clear temporal increase in *D. acuminata* blooms. Our results for *D. acuta* also lead to a similar conclusion for this species.

In 2015 *Dinophysis* in the Firth of Clyde followed the typical pattern of an increase in *D. acuminata* through early summer, followed by a rise in the abundance of *D. acuta* in late summer and early autumn. Our boat-based field survey identified a pronounced temperature front between Stations S6 and S7 near the mouth of Loch Fyne. A substantial drop in nutrient concentrations across the front was also observed [24]. Fronts are known to be associated with dinoflagellate blooms [38,39,40] and in this case, we observed a separation of the phytoplankton community structure into distinct populations on either side of the front [24] with the largest concentrations of *D. acuta* at Stations S7 and S8 to the seaward side of the front (Figure 7), indicating that the front promoted the bloom or at least allowed wind-blown cells to accumulate against it. Such behaviour is consistent with the conceptual model of Smayda and Reynolds [41], who suggest that *Dinophysis* spp. are a transitional life form along the onshore-offshore mixing nutrient gradient, seeming to prefer areas of less pronounced turbulence. While they tolerate coastal upwelling sites and form modest blooms during periods of upwelling relaxation, they are common in areas where there is greater seasonal stratification and lower nutrients. Given that *D. acuta* blooms occurred in the same years in the geographically distinct Clyde Sea and Loch Ewe, local processes do not appear to control the bloom/non-bloom dynamics of this species but are likely to govern the specific location of events and their magnitude. Similar behaviour has also been reported from Irish waters with Raine et al. [42] noting that *D. acuta* mainly occurred in stratified shelf waters in late summer and hypothesizing that *D. acuta* populations developed rapidly close to tidal fronts where productivity is high. It is important to note that *D. acuminata*, although present during our 2015 survey, did not bloom nor aggregate near the frontal region, confirming the species-specific response to environmental forcing of the genus.

Other oceanographic conditions are also thought to be important in influencing *Dinophysis* transport and bloom formation. For example, coastal jets have been linked to movement of *Dinophysis* around the south-west of Ireland [40,43], with blooms being advected into coastal embayments through wind-driven exchange of water masses [40]. Such advection may result in large toxic blooms through physical accumulation rather than in situ growth [10,44]. Particle tracking model simulations by Paterson et al. [24] suggested that *D. acuta* was advectively transported into the Clyde Sea from the open sea of the North Channel. The model also predicted significant interannual differences in advection are likely in the area, with the potential for cells to be more readily exchanged between the outer waters of the Firth of Clyde and the enclosed waters of Loch Fyne (where aquaculture is concentrated) in the absence of a front.

### 3.2. Dinophysis and Toxin Profiles

Our results demonstrate that transition from a *D. acuminata* to a *D. acuta* dominated community in both the Clyde and Loch Ewe occurred on a number of occasions and influenced OA group toxicity detected in a range of bivalve shellfish species collected during the bloom periods. The relationship between *Dinophysis* abundance and accumulation of OA group toxins in shellfish can vary considerably and there are many factors that will determine the amount of toxin ingested by the shellfish and the rate of toxin depuration. These include the availability of other non-toxic phytoplankton species for filter feeding [45], the ingestion of other potentially toxic vector species and the location of the bivalve in the water column, whether suspended from a raft or occupying the intertidal zone [46]. Bivalve contamination will also depend on the response of the shellfish to a specific phytoplankton species or toxin [47,48] and the toxic potential of individual cells ingested, which may vary temporally, geographically and in response to life stage and nutrient availability [2,49,50]. Toxin content can vary within the same species collected from the same location at different times [51,52] and even between cells of a single species within a daily cycle [51]. Different shellfish species are also known to metabolize diarrhetic shellfish toxins differently [4,53] and mussels have been found to accumulate higher levels of diarrhetic shellfish toxins than other species when exposed to the same algal bloom conditions [4,53,54,55,56,57,58]. Consistent with this, the rope-grown mussels obtained from the Firth of Clyde in 2015 showed the highest level of OA group contamination. However, toxins in both Pacific oysters and razor clams remained below the MPL throughout the *Dinophysis* bloom period.

*Dinophysis* has a worldwide distribution but separate species have been associated with the production of different analogues of the OA group toxins, depending on geographic locality. The relative timing and size of *D. acuminata* and *D. acuta* blooms is therefore of considerable significance to the toxic contamination of shellfish that will likely result. Pectenotoxins have been identified as a dominant component of the toxin profile in *D. acuminata* from New Zealand [59], Japan [60,61], Chile [62,63], Argentina [64] and North America [49,63], with smaller amounts of OA and DTX1 also present. In European waters, *D. acuminata* is more associated with the production of OA. Okadaic acid has been linked to *D. acuminata* on the Swedish west coast [65] and in Denmark only OA and OA esters were identified in blue mussels during a bloom of *D. acuminata* [66]. However, a further study on Danish isolates showed strains of *D. acuminata* to produce only PTX2 and no OA or DTX [67]. Further south, OA was identified in *D. acuminata* samples collected in 1998 from north-west Spain and cells obtained in September were found to have an OA concentration of more than double that of June cells [68]. Blooms of *D. acuminata* in Portugal have also been associated with OA [69], as was a bloom in the Bay of Seine (northern France) [70]. Our study is consistent with the geographical patterns described above in demonstrating that *D. acuminata* blooms in Scottish coastal waters are associated with toxin profiles in shellfish dominated by OA, with a smaller contribution from DTX1.

Pectenotoxins also dominated the toxin profile of *D. acuta* from New Zealand waters [59], with a small amount of OA found but no DTX2. By contrast, various studies from around Spain, Portugal and Ireland have associated the presence of *D. acuta* with DTX2. Both OA and DTX2 at an approximate ratio of 3:2 were found in *D. acuta* collected from north-west Spain in 1997 [68] and DTX2, OA and PTX2 were also present in isolates of *D. acuta* from the same area in 2005-6, with a fairly constant ratio of 3:2 for OA:DTX2 [51] but with variations in toxin cell quota in different months [51]. Increased levels of DTX2 in Portuguese shellfish during late summer and early autumn of 1994 and 1995 also were associated with blooms of *D. acuta* [69]. Both DTX2 and OA in varying proportions were found in *D. acuta* obtained from the Celtic Sea and the south coast of Ireland [71,72]. A further study in the same area reported that *D. acuta* related concentrations of DTX2 and PTX2 were greater than OA in all the samples analysed [73]. Further north, during a bloom of predominantly *D. acuta* off the north-east coast of England in September 2002, mussels were found to contain 76% DTX2, 15% OA and 9% PTX2 [74]. Johnson et al. [26] also identified a toxin profile for mussels collected around Great Britain between 2011 and 2015 that contained DTX2 at 63%, with OA at 35% and a small amount of DTX1 (<2%).

Our investigation of the Firth of Clyde 2015 event showed that the proportion of toxins contributing to the OA group varied throughout the year, with more OA and DTX1 detected in shellfish between May and August followed by an increase in DTX2, such that it was the dominant toxin by early September. The increase in the abundance of *D. acuta* and its replacement of *D. acuminata* as the dominant species contributed to a greater proportion of DTX2 contamination of shellfish in both the Clyde in 2015 and Loch Ewe in 2001 and 2002. Some studies have hypothesized that blooms of *D. acuta* may result in extended periods of toxic contamination of shellfish. Vale, 2004 [54], examined the elimination rates of OA and DTX2 in both mussels and oysters and concluded that DTX2 was eliminated at a slower rate than OA in mussels, although the elimination rate of DTX2 in cockles was similar to that of OA. Mussels from Loch Ewe (this study) remained contaminated with both DTX2 and OA into the winter of 2001, although only DTX2 was still present in mussels during winter 2002. In the Clyde mussel samples, the proportion of free OA to esterified OA increased throughout the summer months and DTX2 was predominantly present in non-esterified form. Our study revealed a prolonged period of toxic contamination of shellfish in the absence of any causative organism, due to the continuing presence of DTX2 and, to a lesser extent, OA in mussel and oyster samples through the autumn and winter of 2015 in the Firth of Clyde. These results support the hypothesis proposed by Vale, 2006 [55], that esterified forms of OA and DTX2 in mussels and clams are eliminated more rapidly than free OA and DTX2 but that DTX2 is less esterified than OA in these species, leading to a build-up of free DTX2.

Johnson et al. [26] examined the OA group toxin profiles in 98 samples of mussels obtained from around Great Britain between 2011 and 2015 as part of the official control monitoring programme. They identified three different toxin profiles, the first (GB1 in Figure 8a) being dominated by OA in both free and ester form (about 86%), with a small amount of DTX1 (8%) and very little DTX2 (6%). The second profile (GB2) was also dominated by OA but with a higher proportion of OA in ester form, approximately 59% compared to 36% in GB1. The third profile (GB3) contained a much larger proportion of DTX2 (about 63%) present, mostly as free DTX2 (mean 58%). Pectenotoxins were absent in all the mussel samples tested. Similarly, in an analysis of toxin profiles from various shellfish species obtained around the coast of Scotland, England and Wales between 2011 and 2016, Dhanji-Rapkova et al. [16] identified three distinct profiles, with Profile 1 being dominated by OA (87%), Profile 2 more of a mix between OA (45%) and DTX2 (52%) and Profile 3 mostly composed of DTX2 (85%). A comparison between the toxin profiles in mussels determined from the Johnson et al. [26] investigation and the results obtained in the Firth of Clyde 2015 study (Figure 8) showed that the toxin profiles obtained for mussels in June and July were very similar to the profile for GB2. Okadaic acid contributed >80% to the total toxin content, with approximately twice as much OA in ester form compared with free OA. However, the September and October toxin profiles showed a much greater similarity with the profile for GB3 where DTX2 in both free and ester form contributed >63% to the total toxic potential.

Food business operators are required through regulation to ensure that unsafe products are not placed on the market and a number of relatively simple, rapid and inexpensive kits are commercially available that are used by the shellfish industry to test for the presence of DSTs in harvested shellfish. These use a variety of methods including quantitative enzyme linked immunosorbent assays, protein phosphatase inhibition assays and qualitative lateral flow immunoassays. The investigation by Johnson et al. [26] examined four of these rapid test kits to compare their performance with the results obtained by the official control method (LC-MS/MS) for a threshold of 80 µg OA eq./kg, which is half the MPL of OA group equivalent toxicity allowed in shellfish tissue. The authors found good agreement in the results for both toxin profiles GB1 and GB2, recorded as either positive or negative for the 0.5 MPL threshold but there was a larger proportion of false negatives for profile GB3 with the majority of the rapid test kits. Generally, the results from the rapid test kits gave a reliable indication of OA group toxin contamination in the mussels but performance was less accurate when there was a larger proportion of DTX2 present.

## 4. Conclusions

Our results confirm that the species diversity within *Dinophysis* populations in Scottish waters can vary interannually. *Dinophysis acuminata* is predominant in Scottish coastal waters and its presence is associated with contamination of shellfish with OA and DTX1, although occasional blooms of *D. acuta* can lead to prolonged contamination with DTX2. In some shellfish production areas, there is often a limited “window of opportunity” for harvesting between site closures due to spring/early summer contamination with paralytic shellfish toxins and those caused by diarrhetic shellfish toxins in mid/late summer. The decision to harvest may be based on a negative result returned by a rapid test kit if the tissue sample contains a high proportion of DTX2, although the regulatory LC-MS/MS test could return a positive result for a similar sample. This can result in expensive product recalls and the associated loss of consumer confidence for all shellfish products. Routine enumeration of *Dinophysis* to species level could provide early warning of potential contamination of shellfish with DTX2 and thus determine the choice of the most suitable kit for effective end-product testing. This would provide better protection to the health of consumers and reduce the potential impact to the shellfish aquaculture industry.

## 5. Materials and Methods

### 5.1. Study Areas

The two study sites are both located on the west coast of Scotland, an area characterised by islands and fjordic sea lochs that provide a sheltered location for much of Scotland’s aquaculture industry. Loch Ewe is a fjordic sea loch on the Scottish west coast (Figure 1a), approximately 12 km long, with a mean depth of around 21 m and a maximum depth of 73 m [75]. It has only a slightly discernible sill at 33 m depth, resulting in it being associated with the more open waters of the North Minch [76,77]. Loch Ewe is part of the Scottish Coastal Observatory (SCObs) operated by Marine Science Scotland (MSS) where multiple physical, chemical and biological parameters are collected on a weekly basis [78]. Further south, the Firth of Clyde is a large fjordic basin with a number of associated sea lochs. It opens into the North Channel of the Irish Sea [79]. The regulatory monitoring points in this region were located on the southern end of the Kintyre peninsula, on the eastern side of the Firth of Clyde near Ayr and further north in Loch Striven and Loch Fyne (indicated by A-E in Figure 1b).

### 5.2. Phytoplankton Samples

Seawater samples were collected from classified shellfish production areas, the sampling method being either a PVC sampling tube or a bucket, depending on the depth of water at each site. The sampling tube allows for the collection of a depth-integrated water sample from 0–10 m. A well-mixed 500 mL (SAMS) or 1 L (MSS) sub-sample of this water was fixed on site, to obtain a final concentration of approximately 1% (SAMS) or 0.5% (MSS) acidic Lugol’s iodine and then returned to the laboratory for analysis. The phytoplankton in a 50 mL sub-sample (detection limit 20 cells/L) was allowed to settle onto the base plate of a chamber for a minimum of 20 h before analysis, following the method described by Utermöhl, 1958 [80]. Cells belonging to the order Dinophysiales were identified and enumerated using inverted light microscopy and subsequent re-analysis of samples allowed assignation of cells to species level where this had not been recorded during the original routine analysis for the regulatory monitoring programme. Phytoplankton data collected through regulatory monitoring by both MSS (1996 to June 2005) and SAMS (September 2005 onwards) were examined to determine the prevalence and seasonality of *D. acuminata* and *D. acuta* in the Firth of Clyde and at the long-term MSS SCObs site in Loch Ewe.

Supplementary phytoplankton data were also available from a research cruise that was conducted between 8–9 September 2015 aboard RV Seòl Mara [24], with sampling taking place at stations around the Clyde Sea and in Loch Fyne (Figure 1b). Oxygen, temperature, salinity and fluorescence were recorded by CTD (SBE 19, Seabird Electronics) and water samples were collected by Niskin bottle into 500 mL opaque plastic bottles and preserved with acidic Lugol’s iodine. Three depths were sampled at each station corresponding to surface, chlorophyll maximum and below the chlorophyll maximum, guided by CTD in-situ fluorescence data. Samples were analysed for the presence of phytoplankton following the Utermöhl method.

### 5.3. Shellfish Samples

Shellfish species collected from around the Firth of Clyde consisted of common mussels (*Mytilus edulis*), Pacific oysters (*Magallana gigas*) and razor clams (*Ensis* spp.) (Table 1). Samples were collected, packaged and transported to the Centre for Environment, Fisheries and Aquaculture Science (Cefas) within validated temperature-controlled containers, in accordance with Cefas guidance and protocols. Enough shellfish, with a minimum of ten organisms per sample, were taken to provide a sample characteristic of the Representative Monitoring Point (RMP) and a minimum weight of 100 g tissue once the shellfish were shucked.

Okadaic acid group toxins present in shellfish tissue were determined using a method validated at Cefas [81] based on that described by the EU Reference Method for lipophilic toxins, using liquid chromatography tandem mass spectrometry (LC-MS/MS). This method has been used for official control monitoring in the UK including Scotland since July 2011 [16]. Aliquots of 2.0 ± 0.01 g shellfish tissue from each sample were subjected to a double extraction each using 9 mL of methanol. Post-centrifugation, 0.2 µm filtered supernatants were subjected to both direct LC-MS/MS analysis and an alkaline hydrolysis step to liberate the esterified OA group toxins [82]. Two Waters, Acquity and Acquity *I*-class, (Waters Ltd., Manchester, UK) Ultra Performance Liquid Chromatographic (UPLC) systems were coupled to Waters Xevo TQ and Xevo TQ-S MS/MS systems respectively. A high pH LC method described by Gerssen et al. [14] was adopted with modifications [81]. Mobile phase A was prepared from 2 mM ammonium bicarbonate adjusted to pH 11 ± 0.2 with ammonium hydroxide. Mobile phase B was 2 mM ammonium bicarbonate in 90% acetonitrile, also adjusted to pH 11 ± 0.2 with ammonium hydroxide. On both systems, a Waters BEH C18 reverse phase UPLC column (2.1 × 50 mm, 1.7 µm) was used in series with a pre-column VanGuard cartridge. The mobile phase flow rate was held at 0.6 mL/min, with column temperatures, run times and injection volumes optimised for the two instruments independently. MS/MS parameters used were those given by Turner and Goya, 2015 [83].

Toxins in shellfish extracts were quantified against calibration solutions prepared from Certified Reference Material (CRM) standards obtained from the Institute of Biotoxin Metrology, National Research Council of Canada (NRCC). Chromatographic retention times together with two Selected Reaction Monitoring (SRM) transitions, optimised for each toxin, were utilised for qualitative identification of toxins, with the primary SRM used for quantitative purposes. Hydrolysed extracts were analysed alongside the unhydrolysed filtrates to enable the quantitation of both free OA group toxins (OA, DTX1 and DTX2) and OA group esters (DTX3s) [82]. Within the regulatory monitoring programme, results are reported as total µg OA equivalent/kg, with a Toxicity Equivalency Factor of 0.6 used for DTX2 [9]. Measurement uncertainty, determined for each shellfish species/toxin analogue combination, is then applied to each quantifiable toxin concentration and used to calculate an overall uncertainty for each total toxic potential estimation. As a precautionary measure, the higher value is used to determine whether the total OA equivalent/kg exceeds the MPL, although actual total OA equivalent/kg values are reported in this study. Okadaic acid group toxin values reported in excess of 400 µg OA eq./kg should be regarded as estimations, as such levels fall outside the operationally-defined linear working range of the instrument.

Additional MSS data on OA group toxins in common mussels were also available for Loch Ewe between 2001 and 2002 [13]. The concentrations of okadaic acid, DTX1 and DTX2 in 80% (*v*/*v*) aqueous methanol shellfish extracts were determined using an API 150EX single quadrupole mass spectrometer, equipped with a TurboIonspray source (Applied Biosystems, Warrington, UK). This was coupled to an Agilent 1100 series HPLC system (Agilent Technologies, West Lothian, UK) comprising of a de-gasser, quaternary pump and autosampler. A reversed-phase column was used for the analysis (Thermo Hypersil C_8_ BDS, 50 × 2.1 mm, particle size 3 µm) with a 10 mm guard cartridge of the same stationary phase. The isocratic mobile phase consisted of 2 mM ammonium formate with 50 mM formic acid in 50% (*v*/*v*) acetonitrile. The flow rate and run time for both analyses were 0.25 mL/min and 10 min respectively. An aliquot of 5 µL was injected onto the analytical column. The mobile phase was directly infused into the mass spectrometer after 1.5 min and the initial mobile phase was put to waste using a switching valve.

## Figures and Tables

**Table 1:** Number of phytoplankton and shellfish samples collected from around the Firth of Clyde between May and December 2015 and used in this study. The number of tissue samples containing total OA group equivalent toxicity exceeding the Maximum Permitted Level (MPL) of 160 µg OA eq./kg is also shown.

Site	Shellfish Species	Phytoplankton Samples	Tissue Samples	OA group Equiv. Toxicity > MPL
(A) Campbeltown Loch	Common mussels	16	28	15
(B) Loch Striven	Common mussels	6	22	9
(C) Loch Fyne: Ardkinglas	Common mussels	19	29	9
(D) Loch Fyne: Otter Ferry	Pacific oysters	19	31	0
(E) Barassie	Razor clams	19	20	0
